# Identification and Characterization of Transcription Factors Involved in Geraniol Biosynthesis in *Rosa chinensis*

**DOI:** 10.3390/ijms232314684

**Published:** 2022-11-24

**Authors:** Jiayao Yu, Xiaoyu Liu, Yifang Peng, Qi Li, Yu Han

**Affiliations:** Beijing Key Laboratory of Ornamental Plants Germplasm Innovation & Molecular Breeding, National Engineering Research Center for Floriculture, Beijing Laboratory of Urban and Rural Ecological Environment, Key Laboratory of Genetics and Breeding in Forest Trees and Ornamental Plants of Ministry of Education, School of Landscape Architecture, Beijing Forestry University, Beijing 100083, China

**Keywords:** *Rosa chinensis*, geraniol biosynthesis, WGCNA, *RcNUDX1* promoter, RcWRKY70

## Abstract

Fragrance is an important characteristic of rose flowers and is largely determined by the terpenes. Rose has a unique NUDX1 (NUDIX HYDROLASES 1)–dependent monoterpene geraniol biosynthesis pathway, but little is known about its transcriptional regulation. In this study, we characterized two China rose (*Rosa chinensis*) materials from the ‘Old Blush’ variety with contrasting aromas. We profiled the volatile metabolome of both materials, and the results revealed that geraniol was the main component that distinguishes the aroma of these two materials. We performed a comparative transcriptome analysis of the two rose materials, from which we identified the hydrolase RcNUDX1 as a key factor affecting geraniol content, as well as 17 transcription factor genes co-expressed with *RcNUDX1*. We also determined that the transcription factor RcWRKY70 binds to four W–box motifs in the promoter of *RcNUDX1*, repressing *RcNUDX1* expression, based on yeast one-hybrid and transient dual-luciferase assays. These results provide important information concerning the transcriptional regulatory framework underlying the control of geraniol production in rose.

## 1. Introduction

Terpenes are an important category of volatiles that contribute to floral fragrances [1]. Terpenoids are composed of basic five-carbon (C5) units and can be classified as monoterpenoids (C10), sesquiterpenes (C15), and diterpenes (C20) based on the number of isoprenoids [2,3]. In plants, terpenes are synthesized mainly through two metabolic pathways: the methylerythritol phosphate (MEP) pathway and the mevalonate (MVA) pathway. Both pathways use isopentenyl diphosphate (IPP) and dimethylallyl diphosphate (DMAPP) as precursors [4,5]. The MEP pathway takes place in plastids (but also involves the cytosol, endoplasmic reticulum, and peroxisome) and synthesizes IPP and DMAPP from the substrate glyceraldehyde-3-phosphate and pyruvate. Under the action of transferase, IPP and DMAPP with different molecular weights are condensed to provide substrates for the biosynthesis of monoterpenes and diterpenes. The MVA pathway in the cytoplasm uses acetyl-CoA as a substrate, followed by the condensation of two molecules of IPP and one molecule of DMAPP to form one farnesyl diphosphate (FPP) molecule, itself used as a substrate to form sesquiterpenoids [6,7,8]. Terpene synthases (TPSs) are the key enzymes that catalyze the biosynthesis of terpenes. Many terpene synthase genes have been cloned and studied since the first linalool synthase gene was identified in fairy fans (*Clarkia breweri*) [9]. The research on terpene biosynthesis remains a hot topic.

Rose (*Rosa hybrida*) is the top ranking cut flower in the flower trade on the basis of acreage, production, and consumption. Gardens exclusively for roses have been made in various parts of the world. Rose is also emerging as a model to clarify the molecular mechanism of floral fragrance in woody plants because of its pleasant flower scent [10,11]. In rose, monoterpene alcohols and other volatile terpene substances are some of the main contributors of rose scent [12,13]. Most terpene compounds (especially monoterpenes) are highly volatile and widely used in essential oil production [14]. A unique terpene biosynthesis pathway was described in rose: The nudix hydrolase RhNUDX1 shows geranyl diphosphate phosphohydrolase activity in vitro and is involved in geraniol biosynthesis [2]. Geraniol, a kind of acyclic isoprenoid monoterpenoid, and its isomer nerol are the main components of rose essential oils and have antimicrobial properties [15]. This special geraniol biosynthesis pathway suggests that a new transcription regulatory mechanism may exist in rose.

Terpene biosynthesis is not only related to key synthases but is also regulated by the activity of transcription factors (TFs) on their downstream target genes. Many TFs involved in the transcriptional regulation of genes involved in the floral aroma have been identified and belong to the bHLH (helix-loop-helix), bZIP (basic leucine zipper), AP2/ERF (APETALA2/ETHYLENE-RESPONSIVE ELEMENT BINDING FACTOR), NAC (NAM, ATAF and CUC), MYB, and WRKY families [16]. Most TFs reported to date that regulate terpenoid metabolism belong to the AP2/ERF, WRKY, and bHLH families. In Arabidopsis (*Arabidopsis thaliana*), the *bHLH* member *MYC2* positively regulates the expression of *TPS11* and *TPS21* by binding to the E–box sequence in their promoter regions [17]. In sweet wormwood (*Artemisia annua*), *AaERF1* and *AaERF2* are two positive regulators of artemisinin biosynthesis [18]. *ZmEREB58* isolated from maize (*Zea mays*) can bind to the promoter of *ZmTPS10* and affect the accumulation of farnesene [19]. *LcERF19* from aromatic litsea (*Litsea cubeba*) and *CitERF71* from citrus (*Citrus sinensis*) positively regulate geraniol biosynthesis [20,21]. However, the transcription factors that regulate geraniol biosynthesis in rose have not been reported.

Flower aroma is an important ornamental trait of rose that greatly improves its garden value and is a major focus in rose breeding. With the continuous development of omics technologies, we can now dissect how the floral aroma phenotype is manifested. The *Rosa chinensis* ‘Old Blush’ variety has made important contributions to modern rose cultivars, and a high-quality genome assembly was made available [22]. In this study, we employed two ‘Old Blush’ materials (typical ‘Old Blush’, OB, with strong aroma; ‘Old Blush’–like, OBL, with weak aroma) with significant differences in aroma. We characterized the differentially expressed genes and differentially abundant volatiles between these materials, which revealed geraniol as the main difference volatile in OB and OBL. We also constructed a yeast cDNA library for *R. chinensis* to identify candidate transcription factors that regulate geraniol biosynthesis. The results generated in this study will help further explore the biosynthetic pathway for geraniol in rose.

## 2. Results

### 2.1. Geraniol Is the Most Significant Differentially Abundant Volatile in Petals between OB and OBL

**Figure 1:**
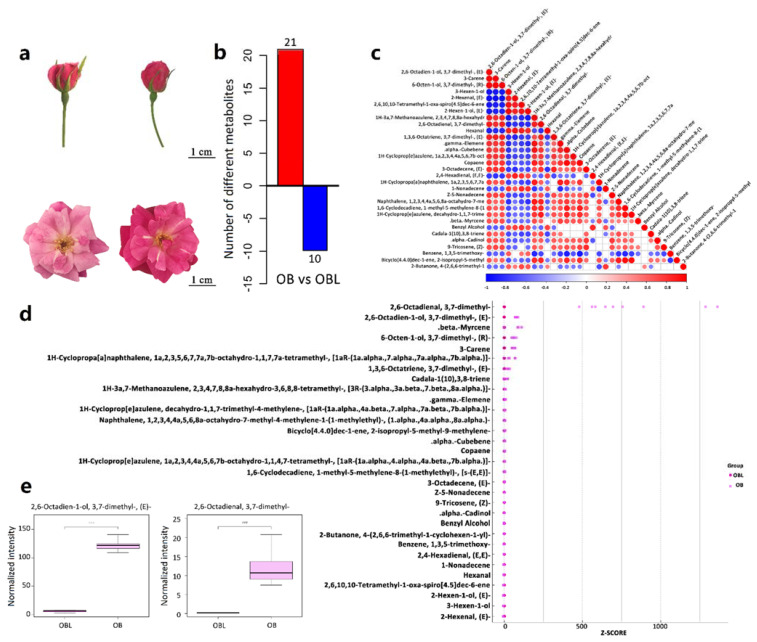
Analysis of differentially abundant volatile compounds between OB and OBL flowers by GC–MS. (**a**) Representative stage 5 flowers (**top**) from ‘Old Blush’ (OB, **left**) and ‘Old Blush’-like (OBL, **right**) used for RNA-seq and GC–MS analyses. Completely open flowers are shown as well (**bottom**). Scale bars, 1 cm. (**b**) Number of differentially abundant volatiles detected by GC–MS between OB and OBL. (**c**) Correlation matrix between the concentrations of various volatiles among samples. The extent of correlation follows the indicated scale from −1 (blue, negative correlation) to +1 (red, positive correlation). The full names of all volatiles are shown in (**d**). (**d**) Significant differentially abundant compounds, identified based on their Z-score, reordered from (**c**) based on decreasing Log2 (Fold-change between OB and OBL). OB and OBL are shown as pink circles and darker pink circles. (**e**) Normalized abundance of geraniol (2,6-octadien-1-ol, 3,7-dimethyl-, (*E*)−) and citral (2,6-octadienal, 3,7-dimethyl-), shown as boxplots.

Flowers from the two materials OB and OBL have a different smell; the only difference in appearance was that OBL flowers are a darker shade of pink than OB flowers (Figure 1a). We conducted a gas chromatography–mass spectrometry (GC–MS) analysis to investigate changes in floral-scented compounds between OB and OBL. Indeed, we observed significant differences in the volatile profiles of OB and OBL flowers (Figure S1, Table S1). We identified 67 volatiles in OB and OBL samples (Table S2). Using the criteria of *p*-value ≤ 0.05 and variable importance in projection (VIP) ≥ 1, we detected 31 differentially abundant volatiles between OB and OBL, of which 21 had relatively higher contents in OB petals (Figure 1 and Figure S2, Table S3). These 31 compounds comprised 17 terpenes, 6 volatiles derived from fatty acids, 1 carotene-derived volatile, 2 volatiles related to the shikimic acid pathway, and 5 other types of volatiles (Table S4).

Terpenes accounted for 54.8% of the 31 compounds that were more abundant in OB petals (Figure S3a). Terpenes also represented the highest proportion of detected compounds in OB petals (Figure S3b). By contrast, compounds derived from the shikimic acid pathway were more abundant in OBL petals (36.2%), followed by volatiles derived from fatty acids (33.3%), with terpenes only representing 28.5% of all volatiles detected in these samples (Figure S3c). Using a Z-score normalization to identify the most abundant compounds revealed geraniol (2,6-octadien-1-ol, 3,7-dimethyl-, (*E*)−) as the top enriched volatile in OB petals, while the second most abundant volatile was citral (2,6-octadienal, 3,7-dimethyl-) (Figure 1d,e). Based on the GC–MS analysis results above, we conclude that the OB and OBL materials and their petals, in particular, are suitable for studying the transcriptional regulation of geraniol biosynthesis.

### 2.2. RcNUDX1 and Geraniol Biosynthesis

**Figure 2:**
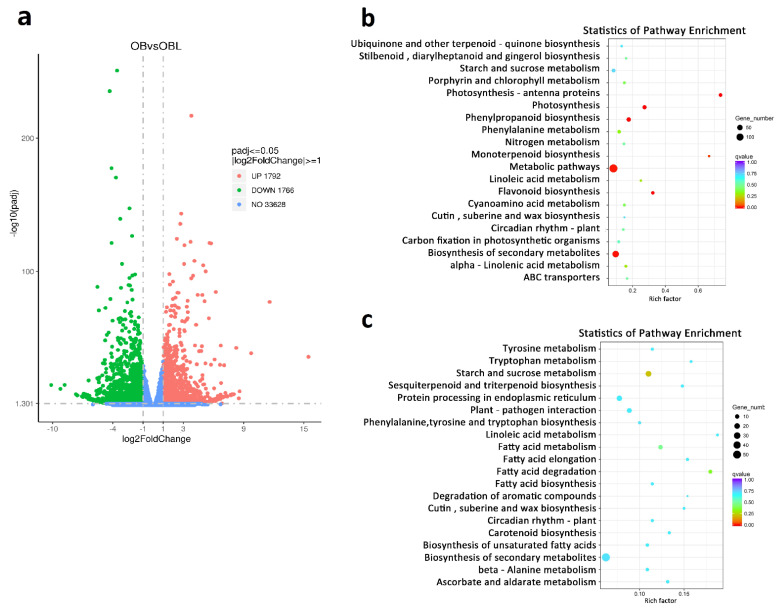
The RNA-seq analysis of stage–5 petals between OB and OBL. (**a**) The volcano map of upregulated (red) and downregulated (green) DEGs between OB vs. OBL. (**b**,**c**) Statistics of KEGG pathway enrichment for upregulated DEGs and downregulated DEGs.

To explore genes related to geraniol biosynthesis, we performed a comparative RNA-seq analysis of petals from OB and OBL flowers using an Illumina HiSeq4000 platform (Tables S5 and S6). We identified differentially expressed genes (DEGs) with the DESeq2 software (v.3.11) in R using the criteria |Log2[OB/OBL Ratio]| ≥ 1 and *p*-value ≤ 0.05. We, thus, obtained 3558 DEGs, with 1766 downregulated genes and 1792 upregulated genes (in OB relative to OBL) (Figure 2a). Kyoto Encyclopedia of Genes and Genomes (KEGG) enrichment analysis indicated that the pathways “metabolic pathways”, “biosynthesis of secondary metabolites”, and “starch and sucrose metabolism” are the most significantly enriched among upregulated DEGs. The most enriched pathway among downregulated genes was “biosynthesis of secondary metabolites” (Figure 2b,c).

**Figure 3:**
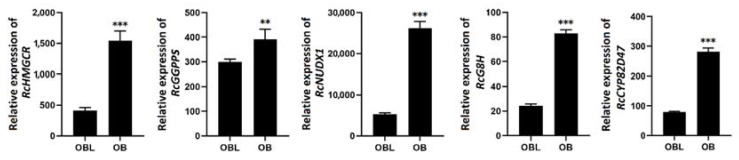
RT–qPCR analysis of geraniol metabolism-related genes. Data are means ± standard deviation of three biological replicates. ** *p* < 0.01, *** *p* < 0.001 (Student’s *t*-test).

To better understand the terpene metabolism pathway of rose, we looked for genes based on functional annotation and sequence homology with related proteins from other plant species. From the first catalytic step executed by 1-deoxy-D-xylulose-5-phosphate synthase (DXS) to the terpene degradation candidate CYP82D47 (cytochrome P450 CYP82D47), we identified 30 members, which are listed in Table S7. Interestingly, only 5 out of these 30 genes were significantly upregulated in OB petals compared to OBL: *RcHMGCR* (encoding hydroxymethylglutaryl-CoA reductase), *RcGGPPS* (encoding geranylgeranyl diphosphate synthase), *RcNUDX1* (encoding a geranyl diphosphate phosphohydrolase), *RcG8H* (encoding geraniol 8-hydroxylase), and *RcCYP82D47*. We hypothesized that RcNUDX1 exerts the same function as RhNUDX1 (GenBank accession number JQ820249), which leads to geraniol production [23]. *RcNUDX1* was present as four tandem copies on chromosome 2 of *R. chinensis* (RchiOBHmChr2g0142071, RchiOBHmChr2g0142081, RchiOBHmChr2g0142111, and RchiOBHmChr2g0142121). The protein encoded by RchiOBHmChr2g0142121 differed by two amino acids compared to the other three NUDX1 copies. We measured the gene expression levels of all five genes in OB and OBL petals by RT–qPCR: *RcNUDX1* was the mostly highly expressed (Figure 3).

### 2.3. Prediction of RcNUDX1 Co-Expressed Genes Based on Weighted Gene Co-Expression Network Analysis

**Figure 4:**
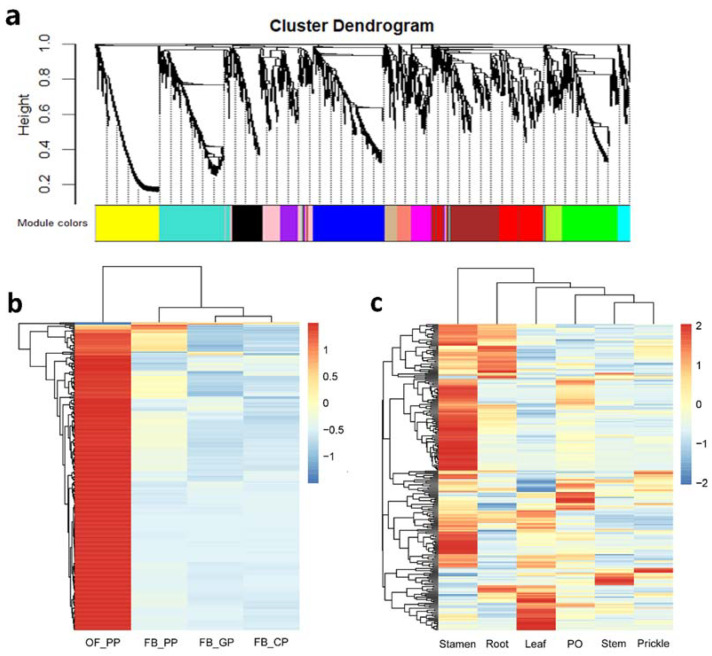
WGCNA of the geraniol biosynthesis-related module. (**a**) Hierarchical cluster tree showing the co-expression modules identified by WGCNA. Each tree leaf represents one gene. (**b**) Heatmap representation of gene expression levels for green-module genes differentially expressed during the following four petal developmental stages of Old Blush: FB_GP, green petals in the flower bud; FB_CP, petals changing colors in the flower bud; FB_PP, pink petals in the flower bud; OF_PP, pink petals on the open flower. (**c**) Heatmap representation of gene expression levels for green-module genes differentially expressed in other tissues of Old Blush plants (stamen, root, leaf, pistil and ovary [PO], stem, and prickle).

To identify genes potentially associated with geraniol biosynthesis, we employed weighted gene co-expression network analysis (WGCNA) using RNA-seq data obtained from the petals of OB and OBL, petals from four different stages of OB, and different tissues of OB. To reduce noise, we only included upregulated genes in OB petals relative to OBL petals, resulting in a set of 3574 genes subjected to WGCNA (Table S8). We detected no outliers in the dataset, based on an analysis of sample clustering (Figure S4a). We set the soft-thresholding power for the analysis of the network topology to 12 (Figure S4b,c). We identified 14 distinct co-expression modules, each labeled with a different color (Figure 4a and Figure S4d,e). The network heatmap plot reveals how the expression of each gene correlates with every other gene (Figure S5).

There were three of the *RcNUDX1* genes in the green-module, which caught our attention. To better represent the expression characteristics of genes within the green-module, we generated a heatmap to represent the expression levels of green-module genes at four stages of petal development and different tissues in OB (Figure 4b,c, Tables S9 and S10). The green-module genes were highly expressed in OF_PP (pink petals when the flowers have just fully opened). In addition to petals, most green-module genes were highly expressed in stamens, which together with petals are used as the main fragrance releasing tissue. Among the 258 genes of the green-module, 17 were annotated as encoding TFs from the MYB, bHLH, bZIP, C2H2, and ERF families.

### 2.4. RcWPKY70 May Be a Transcriptional Repressor of RcNUDX1 Expression

**Figure 5:**
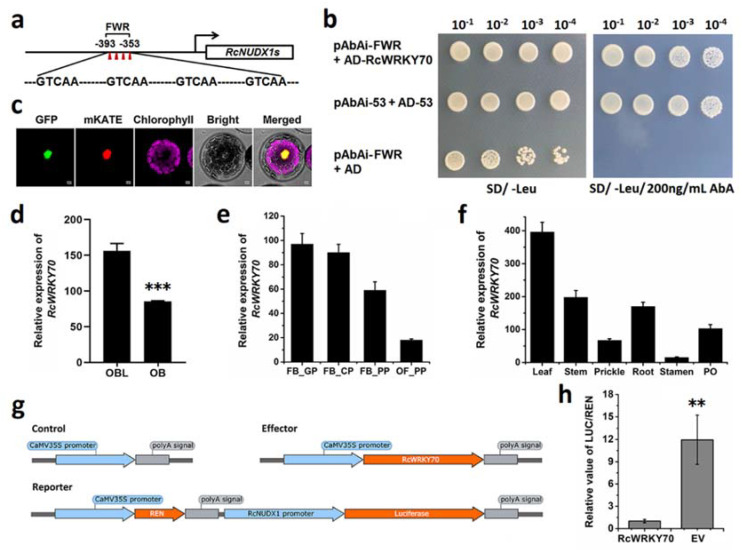
RcWRKY70 binds to and inhibits *RcNUDX1* promoter activation. (**a**) Schematic diagram of the region of four W–box tandem repeats (FWR) in the *RcNUDX1* promoter. (**b**) RcWRKY70 binds to the FWR regions, as shown by Y1H assays. pAbAi–53 + AD–53 represents the negative control; pAbAi–FWR + AD–RcWRKY70 represents the positive control. (**c**) Subcellular localization of *RcWRKY70* in *Arabidopsis* protoplasts. mKATE served as nuclear marker. Scale bars, 10 µm. (**d**–**f**) RT–qPCR analysis of *RcWRKY70* expression. OB, stage 4 petals of Old Blush, OBL, stage 4 petals of Old Blush-like. FB_GP, green petals in the flower bud; FB_CP, petals changing colors in the flower bud; FB_PP, pink petals in the flower bud; OF_PP, pink petals on the open flower. PO, pistil and ovary of ‘Old Blush’. Data are means + SD of three biological replicates. *** *p* < 0.001 (Student’s *t*-test). (**g**) Schematic diagrams of vectors used in the dual–LUC assay. (**h**) In vivo interactions between RcWRKY70 and the *RcNUDX1* promoter as tested by transient assays in *N. benthamiana* leaves. EV, empty vector (Control). Data are means ± SE of three biological replicates. ** *p* < 0.01 (Student’s *t*-test).

We analyzed the DNA sequence characteristics of the *RcNUDX1* genes using the OB genome sequence as a reference [22]. Aside from exhibiting the same exon–intron structures, these *RcNUDX1* genes also possessed the same sequence in a part of their promoter regions (Figure S6), which may have arisen during gene duplication. In this conserved promoter region, we detected four W–box tandem repeats (FWR, Figure 5a).

Therefore, we generated a yeast one-hybrid (Y1H) cDNA library of *R. chinensis* OB and performed a Y1H assay to screen TFs that might bind to the FWR. A concentration of 200 ng/mL of Aureobasidin A (AbA) was sufficient to reduce background in yeast cells harboring the pAbAi–FWR vector (Figure S7). After an initial screening of the cDNA library, we confirmed each positive clone by testing their interaction with pAbAi–FWR again. Sequencing of all positive clones revealed one TF gene, which we named *RcWRKY70* (Figure 5b). We determined that RcWRKY70 localizes to the nucleus, as evidenced by the localization of a RcWRKY70-GFP (green fluorescent protein) fusion and co-localization with the nuclear marker protein mKATE (Figure 5c). To further understand the relationship between *RcWRKY70* and *RcNUDX1*, we investigated their expression levels by RT–qPCR. As shown in Figure 5d,f, *RcWRKY70* was highly expressed in OBL petals and downregulated with the progression of petal development. *RcWRKY70* showed its lowest expression in stamens. The expression pattern of *RcNUDX1* was published [22] and was almost completely opposite that of *RcWRKY70*. To test if RcWRKY70 may regulate expression of *RcNUDX1*, we conducted a dual-luciferase assay in Nicotiana benthamiana leaves. Indeed, the expression of *RcWRKY70* repressed *RcNUDX1* transcription (Figure 5g,h). These results indicate that RcWRKY70 can bind to and repress *RcNUDX1*.

## 3. Discussion

Geraniol is one of the most important components that defines rose fragrance, making geraniol “a pleasant rose-like aroma” chemical [24]. Geraniol has many important biological properties, for example, as an insect repellent [25], an antimicrobial agent [26], and an anti-inflammatory agent [27]. Research on the biosynthesis of natural geraniol could contribute to the full utilization of this substance.

**Figure 6:**
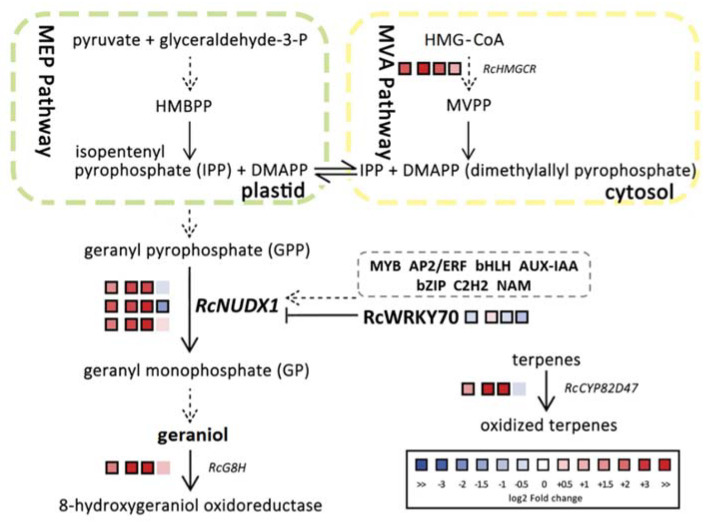
Proposed model of transcriptional regulation of the geraniol metabolic pathway in *R. chinensis*. Each row of the heatmap from left to right represents Log2 (Fold-change) in different developmental stages (FB_CP vs. FB_GP, FB_PP vs. FB_CP, and OF_PP vs. FB_PP) and different samples (OB vs. OBL). The color of each box indicates downregulation (blue) or upregulation (red). Bold margins identify significant differences (*p* ≤ 0.05) between treatments. MVPP, mevalonate-5-pyrophosphate; HMBPP, (*E*)-4-hydroxy-3-methyl-but-2-enyl pyrophosphate; HMG–CoA, 3-hydroxy-3-methylglutaryl-CoA; NUDX, nudix hydrolase; TPS, terpene synthase; G8H, geraniol 8-hydroxylase. Transcript levels for each gene are reported in Table S7.

The typical terpene biosynthetic pathway has been largely elucidated, and the expression and transcriptional regulation of the genes encoding the enzymes acting at each step have been a research hotspot [28]. Rose has a unique geraniol biosynthesis pathway that includes RhNUDX1, an enzyme with geranyl diphosphate diphosphohydrolase activity in vitro that supports geraniol biosynthesis in rose [29]. NUDX1 in rose can convert GPP to GP, thereby participating in geraniol biosynthesis. We obtained two kinds of ‘Old Blush’ with different aromas. Metabolomic analysis revealed that geraniol was the main differentially abundant volatile accumulating in OB petals, making OB flowers a good test material for research on the regulation of geraniol biosynthesis. RNA-seq data indicated that not all genes in the geraniol biosynthesis pathway were upregulated in OB. Indeed, only *RcHMGCR*, *RcGGPPS*, and *RcNUDX1* and two geraniol derivatives-related genes were upregulated in OB relative to OBL. We propose the following model to explain the differences in transcript levels between OB and OBL in the geraniol metabolic pathway (Figure 6). HMGCR catalyzes the NADPH-dependent biosynthesis reaction from HMG-CoA to MVA; since the generation of MVA is irreversible, HMGCR is thought to be the first rate-limiting enzyme in the MVA pathway [30]. Three IPP molecules and one DMAPP molecule form GGPP with a C20 skeleton via catalysis by GGPPS [31]. Geraniol is an acyclic monoterpene alcohol with a C10 skeleton, and its biosynthesis occurs mainly in the plastids and belongs to the MEP pathway. The higher expression of *RcHMGCR* and *RcGGPPS* in OB petals did not appear to have a direct effect on the increase in geraniol content. We speculate that the level of geraniol is directly related to the expression of *RcNUDX1*.

Considering the specificity of the accumulation and release sites of volatiles, we performed WGCNA using published RNA-seq data from OB flowers and the data obtained in this study. We determined that one co-expression module, the green-module, included *RcNUDX1* and several geraniol-biosynthetic genes, whose expression profile closely followed that of geraniol accumulation in OB flowers [22]. Among the 17 co-expressed TF genes in this module, AP2/ERF, NAC, bHLH, and MYB members have been reported to regulate terpene biosynthesis [32,33,34,35]. Other TFs also have connections with terpene-related biological functions; for example, C_2_H_2_ TFs participate in biotic/abiotic stresses responses [36], and bZIP TFs are responsible for plant immunity and abiotic stress responses [37]. However, the possible relationship between RcNUDX1 and the remaining TFs is not clear. These co-expressed TF genes lay the foundation for further studies on the transcriptional regulation of geraniol biosynthesis. We suggest these TFs as candidates for follow-up studies on the transcriptional regulation of geraniol biosynthesis in rose.

TFs are DNA-binding proteins that activate or repress transcription [38]. In addition to activating gene expression, some TFs can also inhibit the expression of target genes. The W–box is the cognate cis-element for WRKY TFs [16]. Some WRKYs were shown to be involved in the transcriptional regulation of plant terpene biosynthesis [39]; the green-module contained no WRKY members. However, the presence of the four tandem W–box repeats suggested some connection between WRKY and RcNUDX1 in rose. We showed here that RcWRKY70 can bind to the *RcNUDX1* promoter and inhibit *RcNUDX1* transcription (Figure 5). Notably, the promoter of several tandemly repeated *RcNUDX1* genes shared a sequence, to which the TF RcWRKY70 bound. We speculate that *RcNUDX1* retained a part of the same promoter sequence (conserved promoter, Figure S6) during gene duplication, which helped maintain the *RcNUDX1* expression pattern. Recent studies have found that the AP2/ERF TFs LcERF19 and CitERF71 can positively regulate geraniol biosynthesis in aromatic litsea and citrus respectively [20,21]. There are still no reports on the transcriptional regulation of WRKY involved in geraniol synthesis. Furthermore, there are few reports of TFs that regulate *NUDX/NUDT* genes. In Arabidopsis, ERF1 binds to the *AtNUDT7* promoter, and *ERF1* is co-expressed with *AtNUDT7* in response to ozone stress [40]. By contrast, *RcWRKY70* and *RcNUDX1* expression levels were negatively correlated. RcWRKY70 can inhibit *RcNUDX1* expression and, thus, geraniol biosynthesis. *WRKYs* are not constitutively expressed in plants but instead are induced by various environmental stresses, such as pathogens, drought, low temperature, or mechanical stress. WRKYs also participate in various physiological processes in plants [41,42,43]. RcWRKY70 may serve as an entry point to study the association between geraniol biosynthesis and other biological events. In the breeding of roses based on floral volatiles, not only a good scent but also the biological characteristics of the volatiles themselves must be considered. Here, we proposed a brief model of the transcriptional regulation of the geraniol metabolism pathway in *R. chinensis* to provide support for molecular breeding of rose fragrance.

## 4. Materials and Methods

### 4.1. Plant Materials and Sample Collection

*R. chinensis* plants were grown in the artificial climate greenhouse at Beijing Forestry University (Beijing, China, located at 116°20′ E and 40°0′ N) under a 25 °C day/18 °C night temperature regime and a 12-h-light/12-h-dark photoperiod with 60% relative humidity. Rose petals were collected at stage 5 (preliminarily opened flower) from 3-year-old *R. chinensis* ‘Old Blush’ (OB) and *R. chinensis* ‘Old Blush’-like (OBL, a natural mutant of OB, similar to OB in terms of phenotypes except for aroma and petal color). Ten healthy rose plants each of OB and OBL were prepared for sampling. All samples were frozen immediately in liquid nitrogen and stored at −80 °C until use.

### 4.2. Gas Chromatography—Mass Spectrometry (GC–MS) Analysis

All stage 5 petals of OB and OBL from three flowers were pooled as one sample; eight biological replicates were collected for GC–MS analysis. The samples were ground to powder, and 0.5 g of powder was quickly transferred into a 20-mL headspace bottle and capped before placing the bottle on a Tray2 tray in a CTC auto-sampler. The needle from the headspace vial completed the solid phase microextraction automatically and inserted the gas extract into the injection port, with the settings 40 min at 80 °C during extracting and 250 °C for 5 min for GC–MS.

The GC system (Agilent 7890A) was coupled to a mass spectrometer (Agilent –5975C Triple Quadrupole, Agilent, Santa Clara, CA, USA). An HB–5MS column (5% phenyl methyl silox: 30 m × 250 µm i.d., 0.25 µm; Agilent J&W scientific, Folsom, CA, USA) was used for all samples. The analytical conditions were set as follows: The initial temperature was held at 50 °C for 1 min and raised to 210 °C for 2 min at 3 °C/min, held for 3 min, and finally, increased to 230 °C at 15 °C/min. GC–MS was conducted in the split-less mode, using helium as the carrier gas with a rate of 1.0 mL/min.

The mass spectrometer was operated in electron-impact (EI) mode at 230 °C, with the electron power set to 70 eV; the auxiliary temperature was set to 280 °C, and the four-stage rod temperature and the quadrupole mass spectrometer were set to 230 °C. The scan range was 80–500 *m*/*z*. The analysis of GC–MS results was carried out as previously described [44]. The Agilent MSD ChemStation software (c.01.08) was used to convert the raw data into netCDF format [45] and annotate each sample using the total peak area normalization algorithm to compare the data of different magnitudes.

### 4.3. Transcriptome Deep Sequencing (RNA-Seq)

Total RNA was extracted using an SV Total RNA Isolation Kit (Promega, Madison, WI, USA) from stage 5 petals following the manufacturer’s instructions. All petals from three flowers for OB or OBL each were pooled as one sample, and three biological replicates were examined. Library construction, sequencing, functional annotation, and analysis of differentially expressed genes (DEGs) were performed according to a previous study [46]. The raw sequencing data were deposited as BioProject PRJNA851232 in the NCBI Sequence Read Archive (National Center for Biotechnology Information). A summary of all reads quantified with StringTie [47] is shown in Table S5. HTSeq v0.9.1 was used to count the number of reads mapped to the rose reference genome (Table S6) [22,48]. Genes with an adjusted *p*-value < 0.05 were considered differentially expressed. Kyoto Encyclopedia of Genes and Genomes (KEGG) enrichment analysis was performed using KOBAS v2.0 (corrected *p*-value < 0.05; http://www.genome.jp/kegg/, accessed on 16 February 2020).

### 4.4. Weighted Gene Co-Expression Network Analysis (WGCNA)

The 3574 upregulated DEGs in OB compared to OBL were used as input for WGCNA (Table S7) [49]. The RNA-seq data from rose petals at different stages of development (BioProject PRJNA351281) and those from different rose tissues (root, stem, leaf, prickle, stamen, pistil, and ovary tissue; BioProject PRJNA546486) were also combined in Table S7 for WGCNA. The removeBatchEffect function in Limma was used to adjust for batch effects [50]. The soft threshold power was calculated using the pickSoftThreshold function in R package WGCNA. Co-expression modules were obtained using the automatic network construction function blockwiseModules of WGCNA with the following settings: power = 12, TOM-Type = unsigned, minModuleSize = 30, and mergeCutHeight = 0.25. The green-module containing RcNUDX1 (RchiOBHmChr2g0142071, RchiOBHmChr2g0142081, and RchiOBHmChr2g0142111) was selected for further study. Heatmaps representing gene expression levels were drawn with the R package pheatmap (Tables S8 and S9).

### 4.5. RT–qPCR Validation

Stage 5 petals and different tissues from OB and OBL were collected and processed for total RNA extraction and first-strand cDNA synthesis as previously described [51]. The key DEGs involved in the terpenoid metabolic pathway and *RcWRKY70* were subjected to validation by RT–qPCR using *RcACTIN* as the reference transcript. Primer sequences are provided in Table S11. qPCR was performed with a Green^®^ Premix Ex Taq™ II (RR420Q, Takara, Tokyo, Japan) and the CFX Connect Real-Time PCR Detection System (Bio–Rad, Hercules, CA, USA). Each sample was tested as three technical replicates. Relative gene expression levels were obtained by the comparative 2^−ΔΔCt^ method [52]. All results were plotted in Origin9 (OriginLab, Northampton, MA, USA).

### 4.6. Subcellular Localization

The coding sequence of *RcWRKY70* was amplified by PCR under the following conditions: 94 °C for 2 min, followed by 35 cycles of 94 °C for 10 s, 60 °C for 15 s, and 72 °C for 2 min, with a final extension of 72 °C for 5 min (KOD–Plus–Neo DNA Polymerase Reaction System, TOYOBO Co., Ltd. Life Science Department, Osaka, Japan). Primer sequences are provided in Table S11. The PCR product was cloned into the pEZS–NL vector (Dr Ehrhardt, Carnegie Institution, Stanford, CA, USA) digested with *Kpn*I and *Xho*I. *A. thaliana* protoplast isolation and transfection were carried out according to a standard protocol [53]. The nuclei were labeled with the fluorescent marker mKATE, with excitation at 588 nm and emission at 635 nm [54]. The transfected protoplasts were incubated at 23 °C for 16 h and observed under a confocal microscope (Leica TCS SP8, Leica Microsystems, Mannheim, Germany) to detect fluorescence; GFP was excited at 488 nm and its emission wavelength was 507 nm.

### 4.7. Cloning of the RcNUDX1 Promoter

Genomic DNA was isolated from ‘Old Blush’ petals using an E.Z.N.A.^®^ Plant DNA Kit (Omega Bio–tek, Norcross, GA, USA) according to the manufacturer’s instructions. A 1207-bp promoter fragment of *RcNUDX1* was amplified using KOD plus DNA polymerase (TOYOBO Co., Ltd. Life Science Department, Osaka, Japan). Primer sequences are provided in Table S11. The cis-motifs in the promoter were analyzed by the online software PlantCARE (http://bioinformatics.psb.ugent.be/webtools/plantcare/html/, accessed on 2 September 2021) and New PLACE (https://www.dna.affrc.go.jp/PLACE/?action=newplace, accessed on 2 September 2021).

### 4.8. Yeast One-Hybrid cDNA Library Creation

Total RNA was separately isolated from *R. chinensis* OB tissues (leaf, flower, stem, and root) using an E.Z.N.A.^®^ Plant RNA Kit (Omega Bio–tek, Norcross, GA, USA) and mixed according to the manufacturer’s instructions. A CloneMiner II cDNA Library Construction Kit (Invitrogen, Carlsbad, CA, USA) was used to generate a cDNA library. The cDNA library was normalized using a Trimmer Direct cDNA Normalization Kit, purified by a TaKaRa MiniBEST DNA Fragment Purification Kit (Takara, Tokyo, Japan), and enriched using a CHROMA SPIN–1000–TE system (Clontech, Mountain View, CA, USA). The enriched cDNA was ligated into the pGADT7–*Sfi*I vector and then the cDNA library was purified with a PureLink™ HQ Mini Plasmid DNA Purification Kit (Invitrogen, Carlsbad, CA, USA).

### 4.9. Construction of Bait Plasmids Construction and Self-Activation Test

The three tandem repeats of the four W–box region (FWR) promoter sequence were synthesized and sequenced (Sunbiotech Co., Ltd., Beijing, China). The FWR was cloned into the pAbAi vector digested with *Hind*III and *Sal*I. Primer sequences are provided in Table S11. The resulting pAbAi–FWR plasmid was digested with *BstB*I and *Bbs*I before integration into the yeast genome of strain Y1H Gold according to the manufacturer’s instructions (Clontech, Mountain View, CA, USA). The bait yeast strains were screened on synthetic defined (SD)-Ura medium with optimal Aureobasidin A (AbA) concentration (0, 100, 150, 200, 300, 500, 700, and 900 ng/mL) to suppress autoactivation after 3 days at 28 °C. The minimum AbA concentration that could completely inhibit strains was determined for library screening.

### 4.10. Yeast One-Hybrid Screening

The full-length *RcWRKY70* coding sequence was cloned into the pGADT7 vector digested with *Nde*I and *BamH*I (primers are listed in Table S11). The Matchmaker™ Gold Yeast One-Hybrid System (Clontech, Mountain View, CA, USA) was used to carry out the Y1H assay according to the manufacturer’s protocol. The yeast cells were spread as 100 μL of 1:10, 1:100, 1:1000, and 1:10,000 dilutions on SD/–Leu plates with 200 ng/mL AbA concentration for 3 days. The positive colonies were tested with Matchmaker Insert Check PCR Mix 2 (Clontech, Mountain View, CA, USA) to amplify prey library inserts. A TIANprep Yeast Plasmid DNA Kit (TIANGEN, Beijing, China) was used to extract the plasmids from yeast, and all clones were sequenced (Sunbiotech Co., Ltd., Beijing, China).

### 4.11. Dual–Luciferase (Dual–LUC) Assay

The full-length *RcWRKY70* coding sequence was cloned into the pGreenII62-SK plasmid (effector) digested with *Xba*I and *Kpn*I, and the *RcNUDX1* promoter fragment was cloned into the pGreenII0800–LUC plasmid (reporter) digested with *Hind*III and *BamH*I. Primer sequences are provided in Table S11. The empty pGreenII62–SK plasmid was used as control for the effector. The effector and reporter were individually transformed into Agrobacterium (*Agrobacterium tumefaciens*) strain GV3101. Agrobacterium cultures grown overnight were collected by brief centrifugation and resuspended in infiltration solution (10 mM MES, 10 mM MgCl_2_, 200 mM acetosyringone, pH 5.8) to an OD_600_ = 1.2 and incubated for 2 h at 23 °C. The cell suspensions were then mixed (1:1, *v*/*v*), harboring the appropriate combination of effector and reporter constructs, and infiltrated into the leaves of 30-day-old *Nicotiana benthamiana* plants with a needleless syringe. Plants were grown at 23 °C for 48 h under a 12-h-day/12-h-dark photoperiod. LUC and REN enzymatic activities were analyzed with a dual-luciferase assay reagent kit (Promega, Madison, WI, USA).

## Data Availability

The raw sequencing data of RNA-seq in this study were deposited as BioProject PRJNA851232 in the NCBI Sequence Read Archive (National Center for Biotechnology Information).

## Supplementary Materials

The following supporting information can be downloaded at: https://www.mdpi.com/article/10.3390/ijms232314684/s1. Figure S1: OPLS–DA, PCA, and PLS–DA of metabolites with permutation test; Figure S2: Hierarchical clustering heatmap of 31 volatiles based on GC–MS results; Figure S3: Comparative analysis of differentially abundant volatiles in OB and OBL petals. (a) Pie charts showing the classification and distribution of differentially abundant volatiles in OB and OBL. (b) Distribution of volatile contents in OB. (c) Distribution of volatile contents in OBL; Figure S4: WGCNA of 3574 DEGs between OB and OBL. (a) A 26-sample clustering analysis to detect outliers. (b) Soft threshold (power) screening. R^2^ = 0.85. (c) Analysis of the soft threshold and mean connectivity. (d) Branches of the module group together with eigengenes that are positively correlated. (e) Heatmap of the adjacencies in the eigengene network between modules. Red, high adjacency; blue, low adjacency; Figure S5: The heatmap of the Topological Overlap Matrix (TOM) among all genes in WGCNA; Figure S6: Schematic diagram of the distribution of genetic elements from RchiOBHmChr2g0142121 to RchiOBHmChr2g0142051 in the rose genome; Figure S7: Determination of the optimum concentration of AbA for yeast one-hybrid screening; Table S1: The OPLS–DA, PCA, and PLS–DA parameters model for all detected compounds; Table S2: The PLS–DA variable importance in projection scores of 67 compounds; Table S3: The correlation matrix information between 31 compounds; Table S4: List of differential volatile compounds analyzed by GC–MS in petals of OBL and OB; Table S5: Analysis of the read quality for 6 samples. Error (%) represent the error rate of sequenced bases; Table S6: Number of RNA-seq reads sequenced and mapped on the *R. chinensis* genome; Table S7: Functional annotation and classification of DEGs involved in terpene biosynthesis pathway by RNA-seq analysis (OB vs. OBL); Table S8: FPKMs of the 3574 DEGs across 26 samples used for the WGCNA (|log2Ratio| ≥ 0.5 and *p* ≤ 0.05, OB vs. OBL); Table S9: DEGs information of the green-module in four stage petals of *R. chinensis* ‘Old Blush’; Table S10: DEGs information of the green-module in six organs of *R. chinensis* ‘Old Blush’; Table S11: Primer sequences used in this study.
