# Supplementary material for: Identification and Characterization of Transcription Factors Involved in Geraniol Biosynthesis in Rosa chinensis
Source: Int J Mol Sci. 2022 Nov 24;23(23):14684. doi: 10.3390/ijms232314684 (PMC9739587; doi:10.3390/ijms232314684)
Supplement: Supplementary file 1 [file ijms-23-14684-s001.zip › ijms-2003504-SI.pdf]

International Journal of *Molecular Sciences*

MDPI

Article

Identification and Characterization of Transcription Factors Involved in Geraniol Biosynthesis in *Rosa chinensis*

Jiayao Yu †, Xiaoyu Liu †, Yifang Peng, Qi Li and Yu Han *

Beijing Key Laboratory of Ornamental Plants Germplasm Innovation & Molecular Breeding, National Engineering Research Center for Floriculture, Beijing Laboratory of Urban and Rural Ecological Environment, Key Laboratory of Genetics and Breeding in Forest Trees and Ornamental Plants of Ministry of Education, School of Landscape Architecture, Beijing Forestry University, Beijing 100083, China

* Correspondence: 15201425912@126.com

† These authors contributed equally to this work.

Abstract: Fragrance is an essential characteristic of rose flowers and is primarily determined by the terpenes. Rose has a unique NUDX1 (NUDIX HYDROLASES 1)-dependent monoterpene geraniol biosynthesis pathway, but little is known about its transcriptional regulation. This study characterized two China rose (*Rosa chinensis*) materials from the 'Old Blush' variety with contrasting aromas. We profiled the volatile metabolome of both materials, and the results revealed that geraniol was the main component that distinguishes the smell of these two materials. We performed a comparative transcriptome analysis of the two rose materials, from which we identified the hydrolase *RcNUDX1* as a critical factor affecting geraniol content and 17 transcription factor genes co-expressed with *RcNUDX1*. We also determined that the transcription factor RcWRKY70 binds to four W-box motifs in the promoter of *RcNUDX1*, repressing *RcNUDX1* expression based on yeast one-hybrid and transient dual-luciferase assays. These results provide important information about the transcriptional regulatory framework underlying the control of geraniol production in rose.

Keywords: *Rosa chinensis*, Geraniol biosynthesis, WGCNA, *RcNUDX1* promoter, RcWRKY70

Citation: Yu, J.; Liu, X.; Peng, Y.; Li, Q.; Han, Y. Identification and Characterization of Transcription Factors Involved in Geraniol Biosynthesis in *Rosa chinensis*. *Int. J. Mol. Sci.* **2022**, *23*, x. https://doi.org/10.3390/xxxxx

Academic Editor: Zsófia Bánfalvi

Received: 16 October 2022
Accepted: 22 November 2022
Published: 24 November 2022

Publisher's Note: MDPI stays neutral with regard to jurisdictional claims in published maps and institutional affiliations.

Copyright: © 2022 by the authors. Submitted for possible open access publication under the terms and conditions of the Creative Commons Attribution (CC BY) license (https://creativecommons.org/licenses/by/4.0/).

Supplementary Figures

Figure S1: OPLS-DA, PCA, and PLS-DA of metabolites with permutation test;

Figure S2: Hierarchical clustering heatmap of 31 volatiles based on GC-MS results;

Figure S3: Comparative analysis of differentially abundant volatiles in OB and OBL petals;

Figure S4: WGCNA of 3,574 DEGs between OB and OBL;

Figure S5: The heatmap of the Topological Overlap Matrix (TOM) among all genes in WGCNA;

Figure S6: Schematic diagram of the distribution of genetic elements from RchiOBHmChr2g0142121 to RchiOBHmChr2g0142051 in the rose genome;

Figure S7: Determination of the optimum concentration of AbA for yeast one-hybrid screening;

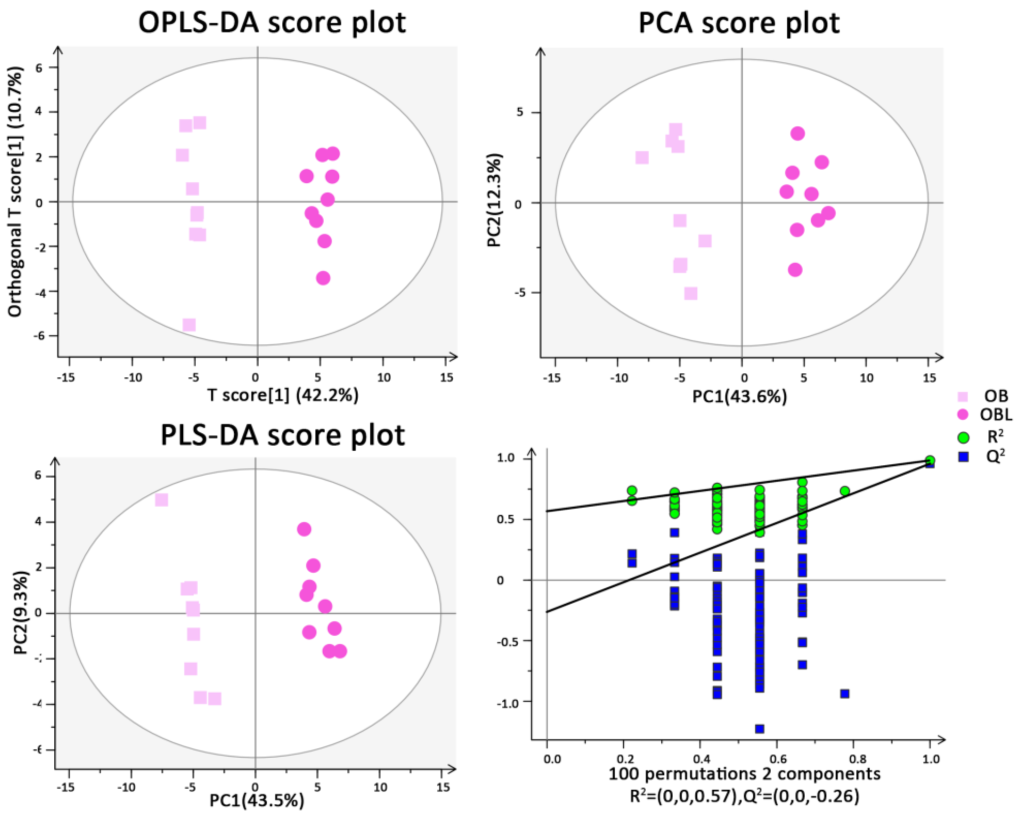

Figure S1. OPLS-DA, PCA, and PLS-DA of metabolites with permutation test. OPLS-DA, orthogonal projections to latent structures discriminant analysis; PCA, principal component analysis; PLS-DA, partial least-squares discriminant analysis. OB, *R. chinensis* 'Old Blush'; OBL, *R. chinensis* 'Old Blush'-like; R2: correlation coefficient; Q2: model prediction ability.

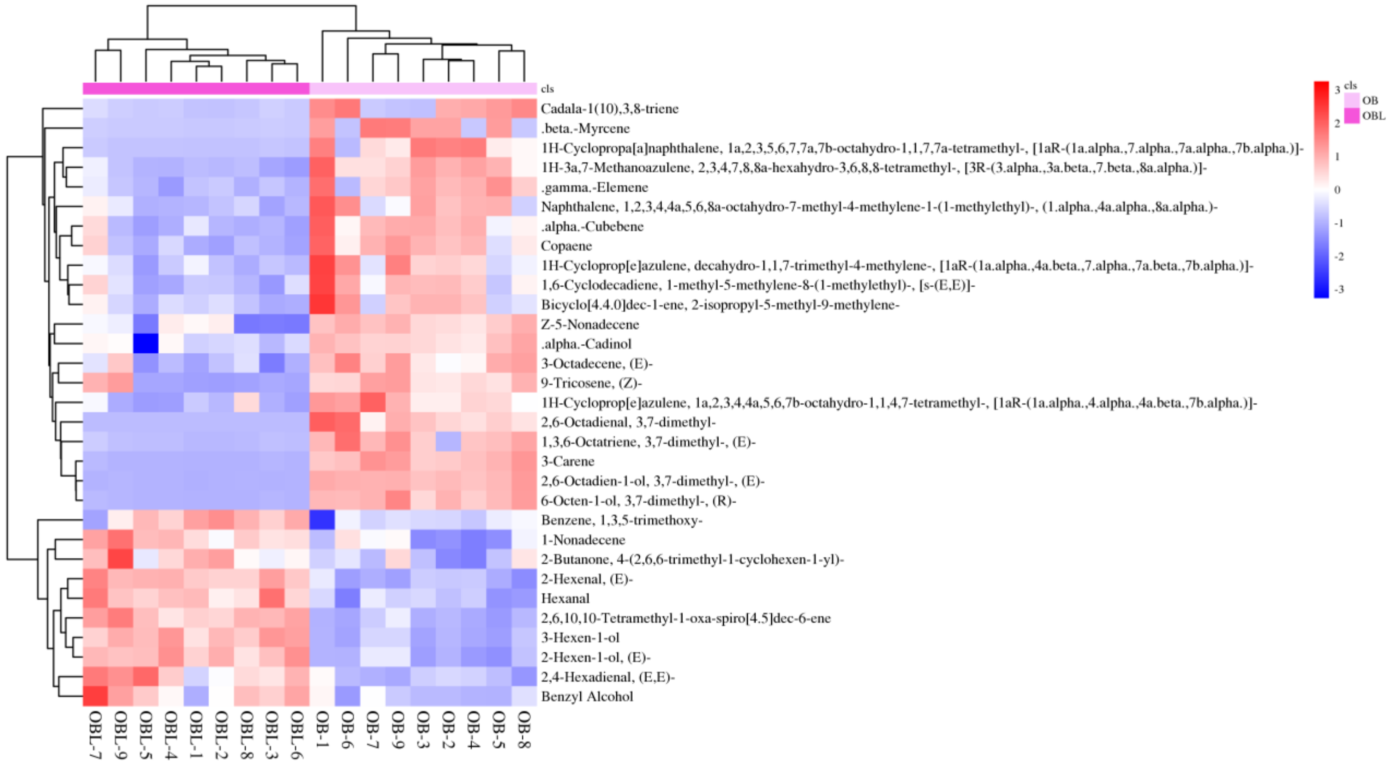

Figure S2. Hierarchical clustering heatmap of 31 volatiles based on GC-MS results.

Each row represents the various samples; each column represents one compound. Blue, low relative content; red, high relative content.

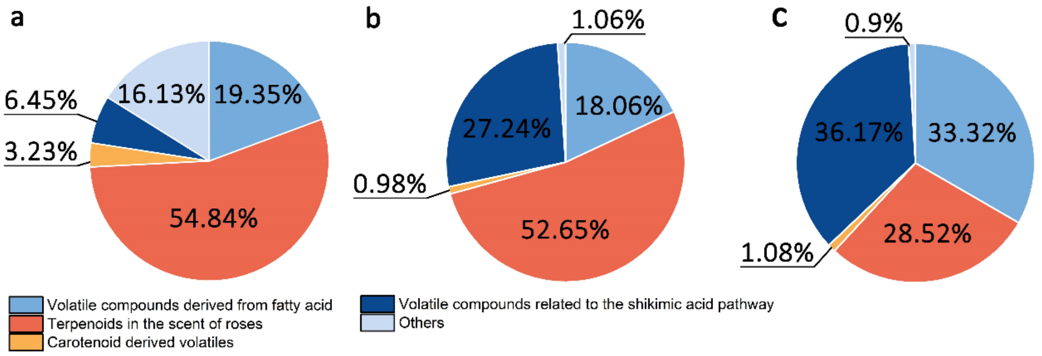

Figure S3. Comparative analysis of differentially abundant volatiles in OB and OBL petals. (**a**) Pie charts showing the classification and distribution of differentially abundant volatiles in OB and OBL. (**b**) Distribution of volatile contents in OB. (**c**) Distribution of volatile contents in OBL.

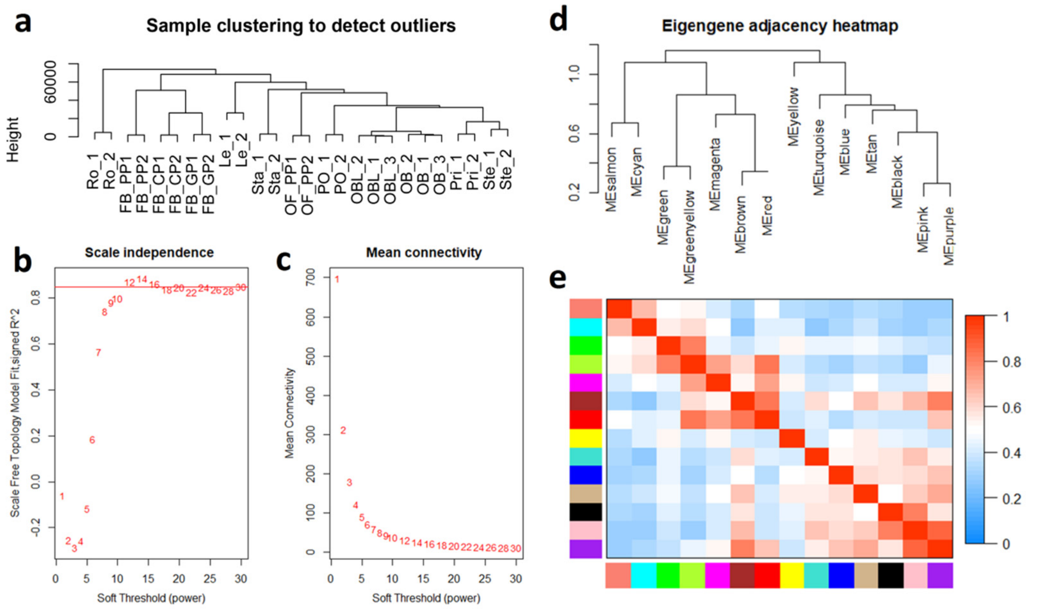

Figure S4. WGCNA of 3,574 DEGs between OB and OBL. (**a**) A 26-sample clustering analysis to detect outliers. (**b**) Soft threshold (power) screening. $R^2 = 0.85$. (**c**) Analysis of the soft threshold and mean connectivity. (**d**) Branches of the module group together with eigengenes that are positively correlated. (**e**) Heatmap of the adjacencies in the eigengene network between modules. Red, high adjacency; blue, low adjacency.

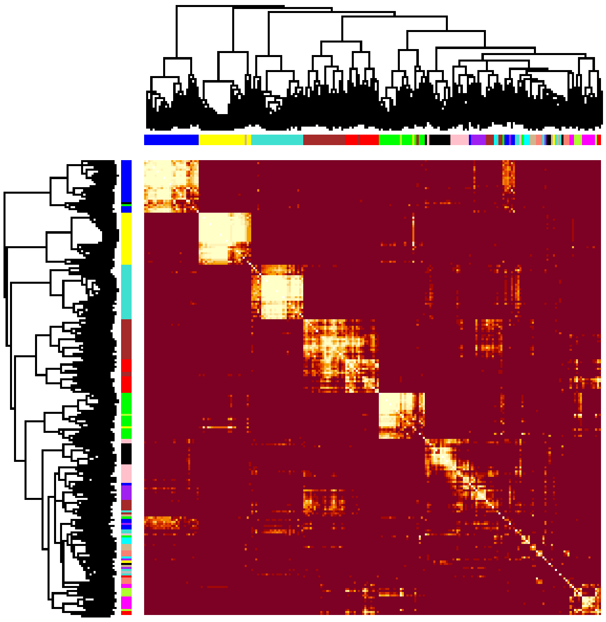

Figure S5. The heatmap of the Topological Overlap Matrix (TOM) among all genes in WGCNA. Red color represents low overlap and light color represents higher overlap.

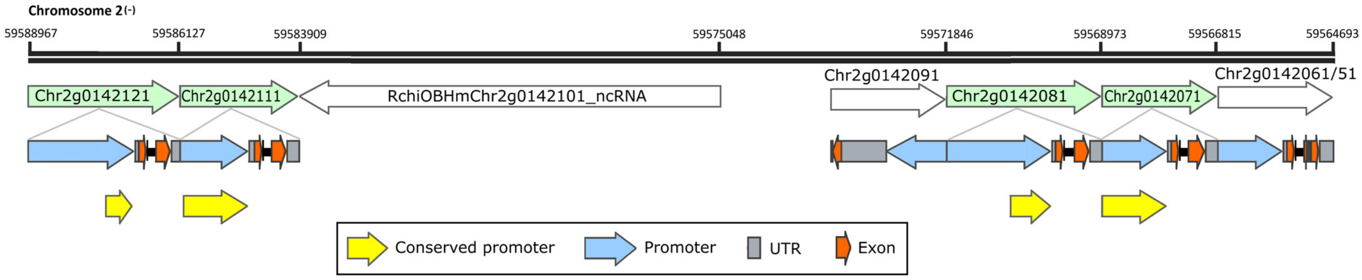

Figure S6. Schematic diagram of the distribution of genetic elements from *RchiOBHmChr2g0142121* to *RchiOBHmChr2g0142051* in the rose genome. The positions of these genes on chromosome 2 of the ‘Old Blush’ genome have been marked with numbers. Four copies of *RcNUDX1* are indicated by green arrows. Their gene structures are marked below: blue arrows represent the promoter regions, orange arrows represent the exon regions, gray arrows represent the UTR regions, and yellow arrows represent the conserved promoter regions;

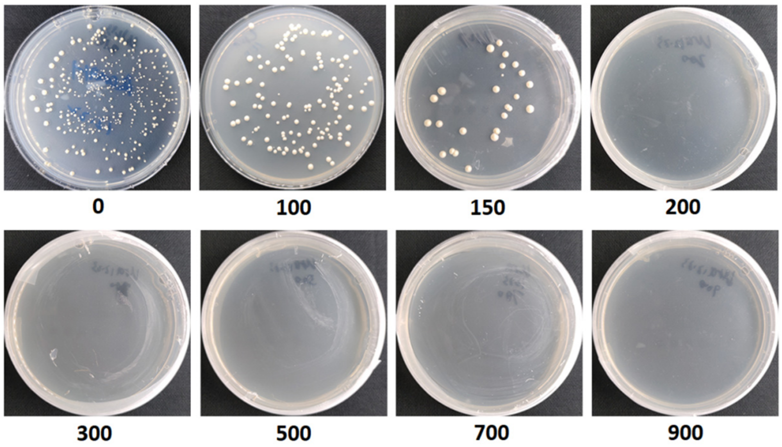

Figure S7. Determination of the optimum concentration of AbA for yeast one-hybrid screening. The unit is ng/mL.

Supplementary Tables

Table S1: The OPLS-DA, PCA, and PLS-DA parameters model for all detected compounds;

Table S2: The PLS-DA variable importance in projection scores of 67 compounds;

Table S3: The correlation matrix information between 31 compounds;

Table S4: List of differential volatile compounds analyzed by GC-MS in petals of OBL and OB;

Table S5: Analysis of the read quality for six samples. Error (%) represent the error rate of sequenced bases;

Table S6: Number of RNA-seq reads sequenced and mapped on the *R. chinensis* genome;

Table S7: Functional annotation and classification of DEGs involved in terpene biosynthesis pathway by RNA-seq analysis (OB vs. OBL);

Table S8: FPKMs of the 3,574 DEGs across 26 samples used for the WGCNA ($|\log2Ratio| \geq 0.5$ and $p \leq 0.05$, OB vs. OBL);

Table S9: DEGs information of the green-module in four stage petals of *R. chinensis* 'Old Blush';

Table S10: DEGs information of the green-module in six organs of *R. chinensis* 'Old Blush';

Table S11: Primer sequences used in this study.
