# Supplementary figures and images for: Identification and Characterization of Transcription Factors Involved in Geraniol Biosynthesis in Rosa chinensis
Source: Int J Mol Sci. 2022 Nov 24;23(23):14684. doi: 10.3390/ijms232314684 (PMC9739587; doi:10.3390/ijms232314684)

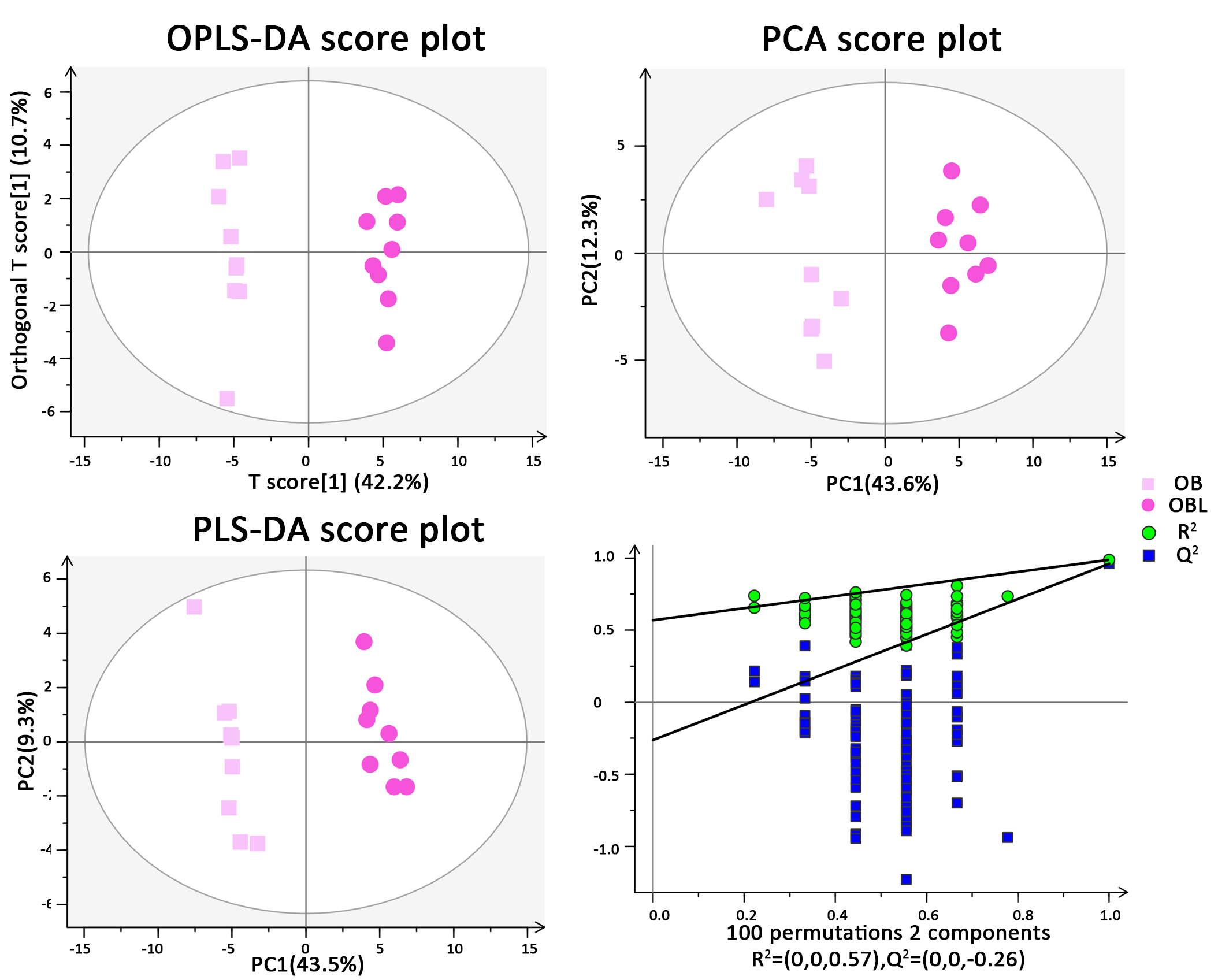

Supplement: Supplementary file 1 [file ijms-23-14684-s001.zip › Figure S1.tif]

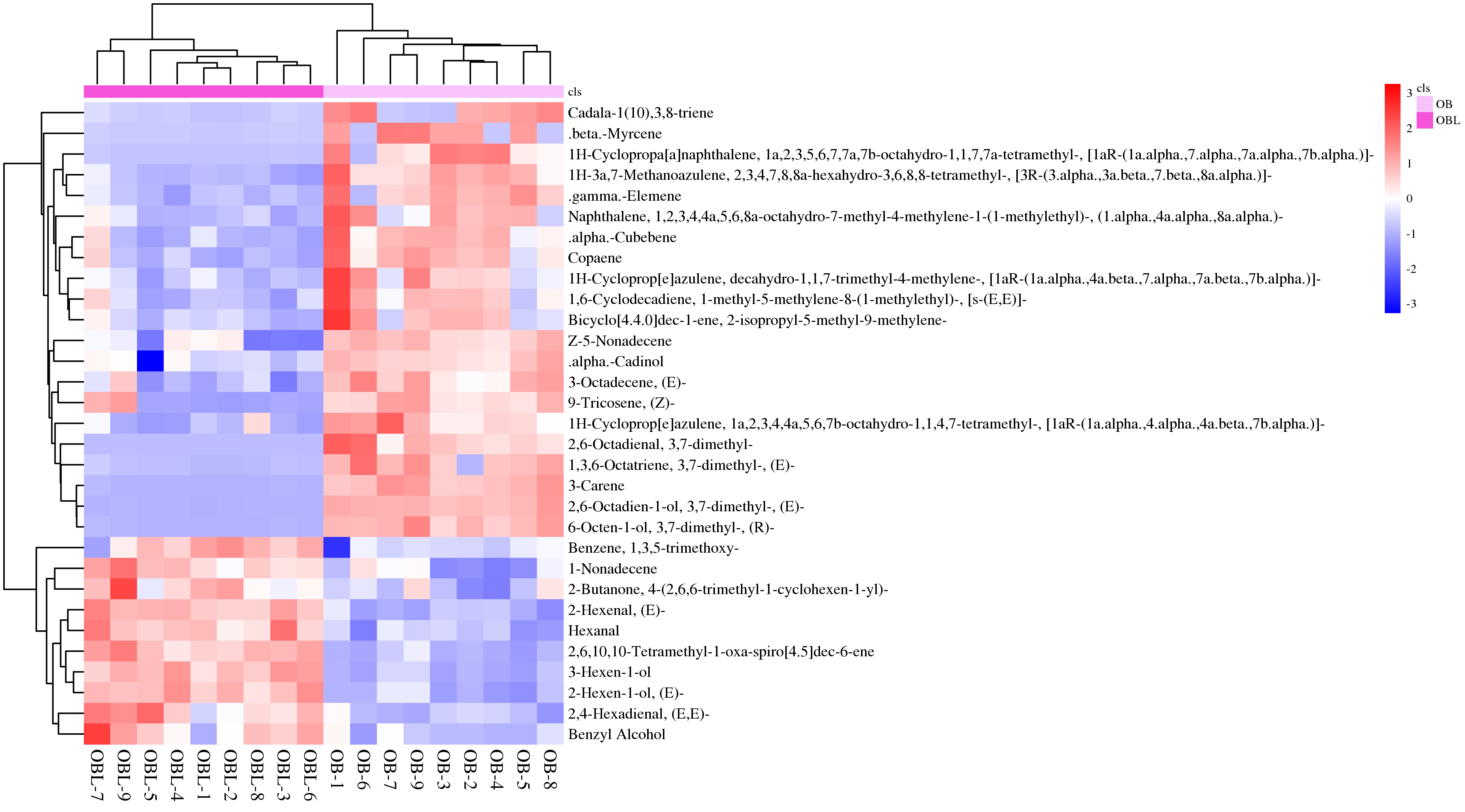

Supplement: Supplementary file 1 [file ijms-23-14684-s001.zip › Figure S2.tif]

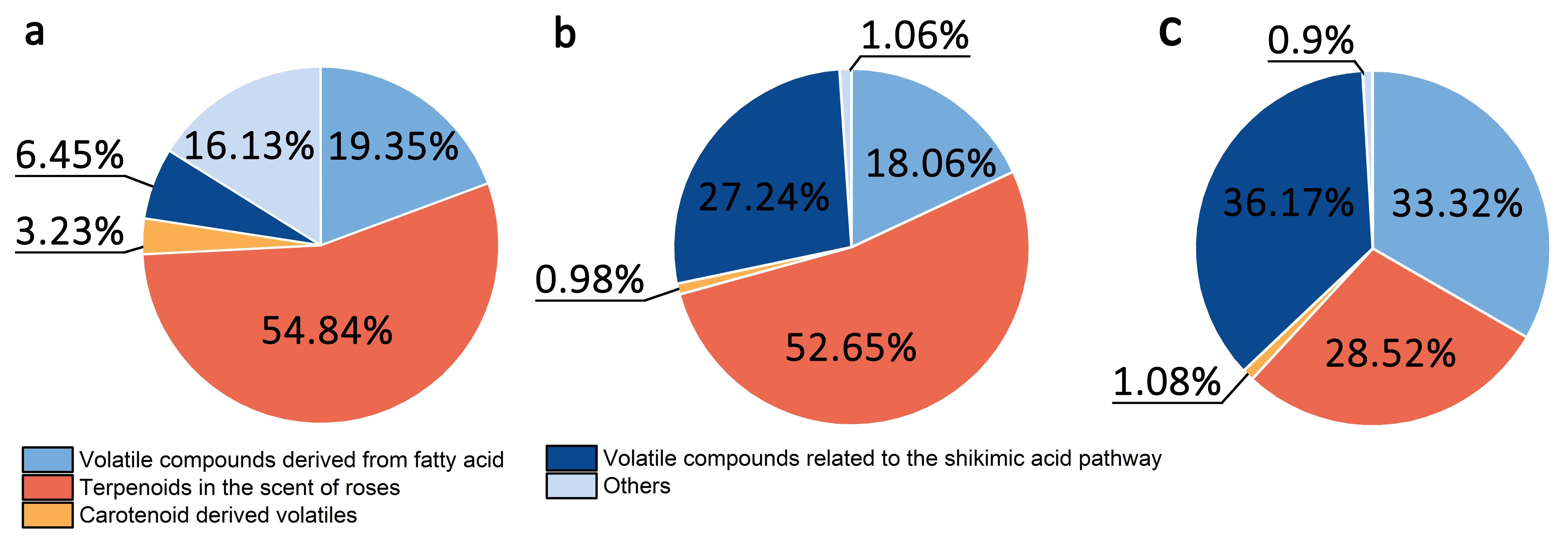

Supplement: Supplementary file 1 [file ijms-23-14684-s001.zip › Figure S3.tif]

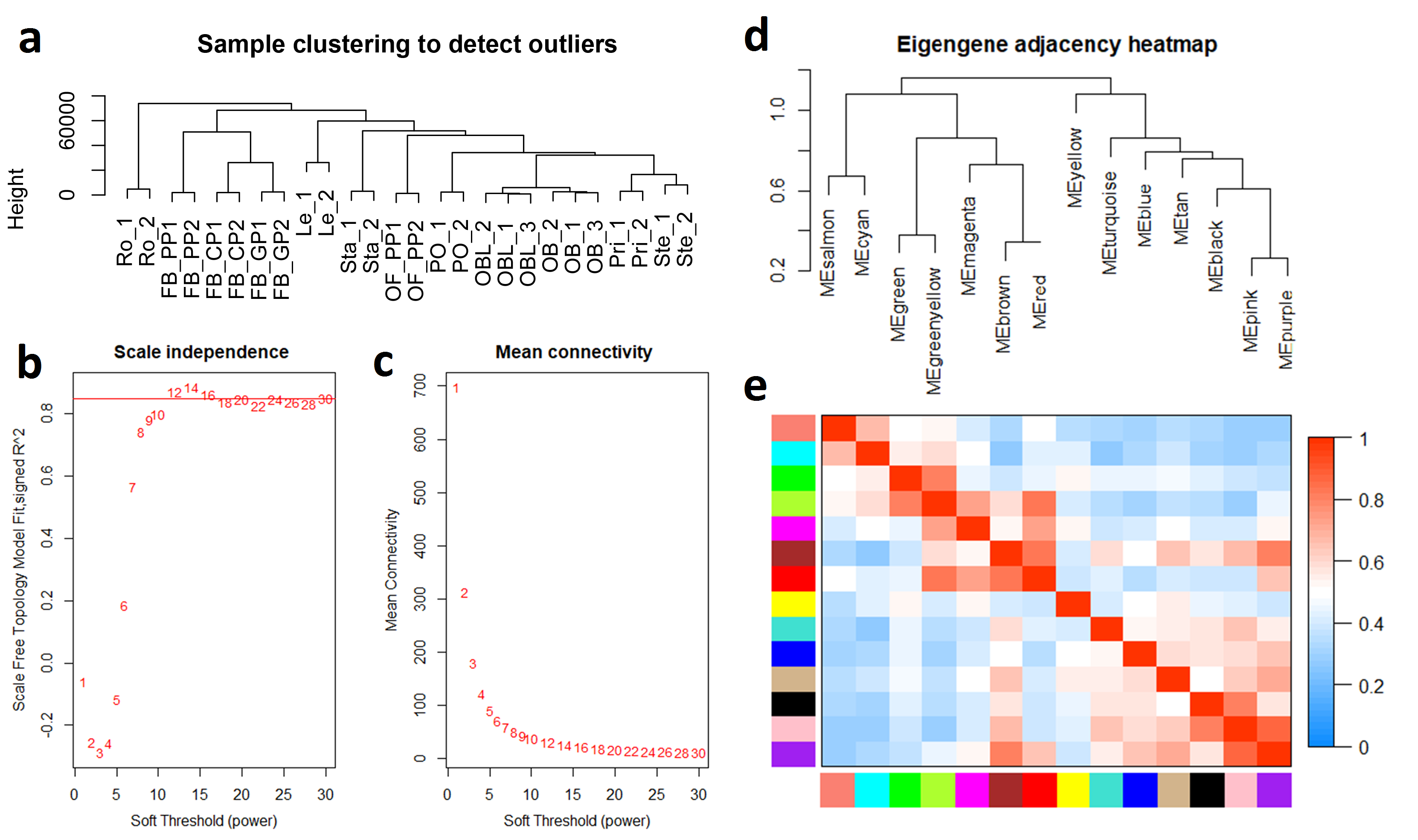

Supplement: Supplementary file 1 [file ijms-23-14684-s001.zip › Figure S4.tif]

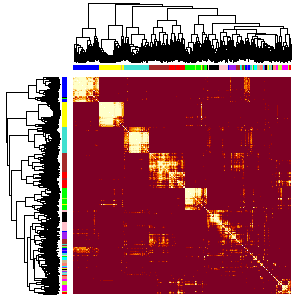

Supplement: Supplementary file 1 [file ijms-23-14684-s001.zip › Figure S5.tif]

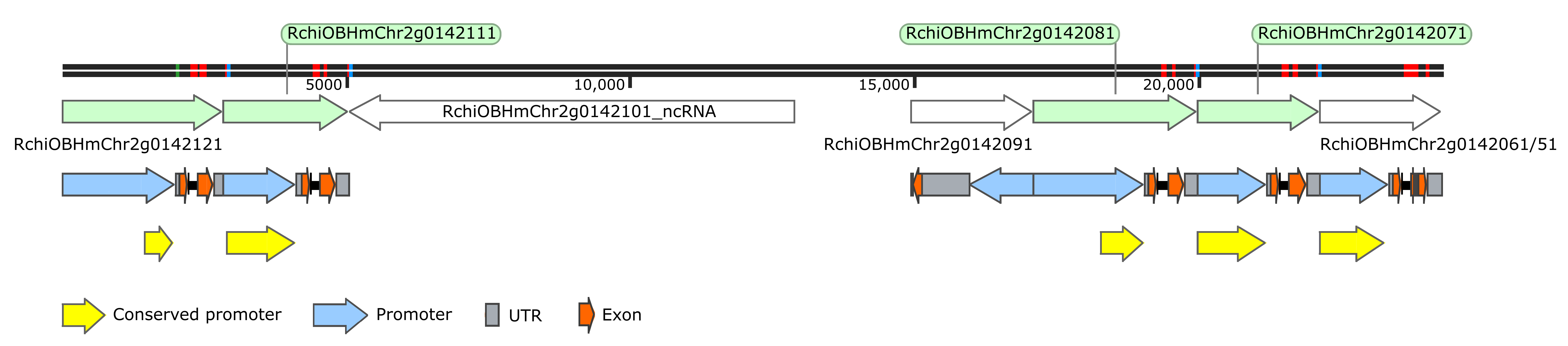

Supplement: Supplementary file 1 [file ijms-23-14684-s001.zip › Figure S6.tiff]

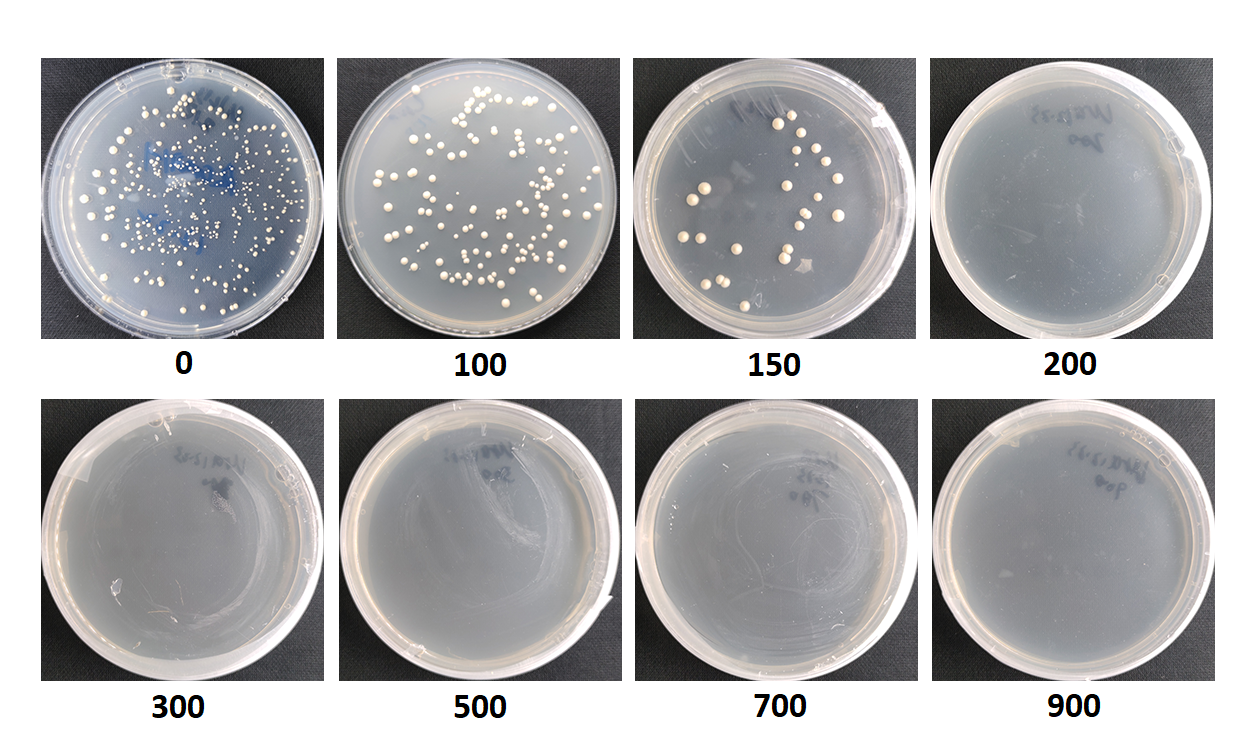

Supplement: Supplementary file 1 [file ijms-23-14684-s001.zip › Figure S7.tif]
